# Controlling the Morphology of Tellurene for a High-Performance H_2_S Chemiresistive Room-Temperature Gas Sensor

**DOI:** 10.3390/nano13192707

**Published:** 2023-10-05

**Authors:** Yeonjin Je, Sang-Soo Chee

**Affiliations:** 1 Nano Convergence Materials Center, Korea Institute of Ceramic Engineering and Technology (KICET), Jinju 52851, Republic of Korea; jejinjin7@gmail.com; 2 Department of Materials Science and Engineering, Korea University, Seoul 02841, Republic of Korea

**Keywords:** 2D materials, tellurene, H_2_S, chemiresistive sensor, room temperature

## Abstract

A two-dimensional (2D) van der Waals material composed only of tellurium (Te) atoms—tellurene—is drawing attention because of its high intrinsic electrical conductivity and strong interaction with gas molecules, which could allow the development of high-performance chemiresistive sensors. However, the correlation between the morphologies and gas detection properties of tellurene has not yet been studied in depth, and few reports exist on tellurene-based hydrogen sulfide (H_2_S) chemiresistive sensors in spite of their strong interaction with H_2_S molecules. Here, we investigate the morphology-dependent H_2_S gas detection properties of tellurene synthesized using a hydrothermal method. To tailor the morphologies of tellurene, the molecular weight of the surfactant was controlled, revealing that a 1D or 2D form was synthesized and also accompanied with the high crystallinity. The 1D tellurene-based chemiresistive sensor presented superior H_2_S detection properties compared to the 2D form, achieving a gas response (R_g_/R_a_) of ~38, even at room temperature. This outstanding performance was attributed to the high intrinsic electrical conductivity and high specific surface area of the resultant 1D tellurene.

## 1. Introduction

Hydrogen sulfide (H_2_S) is a toxic and odorous gas that is mainly generated by the decomposition of plants and animals [1]. H_2_S causes not only severe atmospheric pollution, but also the formation of acid rain [2]. This harmful gas may trigger various symptoms, including headaches, nausea, and irritation to the eyes and respiratory system, in individuals continuously exposed to a certain concentration of it (approximately 10 ppm) [3]. Specifically, it is possible to cause severe pain by stimulating the mucous membranes of the eyes and respiratory tract even at ppm-level exposure. Hence, it is highly essential to establish a H_2_S gas detection system [4]. H_2_S gas-sensing materials are usually metal oxide semiconductors that require a high-operating temperature (>300 °C) [4]; their gas detection mechanism can be described by the changes in the resistance of the surface depletion layers, generated at high temperatures [4]. Such high-temperature operation typically shows a good gas response, but also involves a higher power consumption, which makes it difficult to develop a portable gas-monitoring system [5].

Two-dimensional (2D) van der Waals materials are composed of atomic-layered materials, which significantly represent the high specific surface area. This distinct point can give rise to the strong surface reaction with gas molecules. This interaction allows to achieve low-temperature operation below 100 °C, or even at room temperature, owing to their underlying gas-sensing mechanism; the charge transfer occurs directly between the target gas molecules and the surface of 2D materials [6,7]. This mechanism helps achieve a lower power consumption, which could enable a portable gas-monitoring system to be established, in contrast to metal oxide-based gas sensors [8]. Two-dimensional transition metal dichalcogenides (TMDs), one of van der Waals materials, have been mostly utilized as gas-sensing materials that operate at room temperature [9,10], but they have been significantly challenging to achieve a high gas response and fast operation because of their low-carrier mobilities [10,11]. To overcome this issue, Pham et al. employed the red light-emitting diodes, which can boost the electron carrier mobility of the resultant TMD sensor devices, achieving the higher gas response and the improved speed. Doping or defect-passivation strategy may also be the proper approach to achieve a high gas response and fast operation, but the reliabilities of their sensor devices were significantly poor because it is highly difficult to control their concentration.

Tellurene, a type of 2D van der Waals material, has a narrow band gap (<1 eV) and a distinct chiral-chain crystal lattice with individual helical chains of tellurium (Te) atoms [12]. This anisotropical lattice configuration is similar to 2D black phosphorus. Thanks to this unusual crystal structure, it provides the high air stability, and its field-effect mobility is estimated to be approximately 10^2^ cm^2^/Vs [12]. These unique features make it a good candidate for a high-performance chemiresistive room-temperature gas sensor, contrary to other 2D TMD materials.

There have been various reports on tellurene-based chemiresistive gas sensors devices [13,14,15]. Wang et al. fabricated the nitric dioxide (NO_2_) chemiresistive gas sensors using tellurene flakes obtained through a hydrothermal method. Their resultant devices showed the high gas response of 120% when exposure to 1 ppm of NO_2_ and maintained the stable performance until 30 days [15]. Cui et al. similarly reported tellurene-based NO_2_ chemiresistive gas sensor devices, achieving the superior gas response above 200%, even under exposure to ppb-level concentration. While tellurene-based chemiresistive gas sensor devices have been extensively studied, the majority of the literature has mostly focused on the NO_2_ detection, even though tellurene is also sensitive to H_2_S molecules [16,17]. Furthermore, comprehensive studies on the morphology-dependent gas detection properties of tellurene remain to be reported in detail.

Here, we investigated how the morphology of tellurene is influenced by the molecular weight of polyvinylpyrrolidone (PVP) in the hydrothermal synthesis. PVP is a conventional surfactant [18], resulting in different morphologies of samples depending on its molecular weight. Finally, we fabricated tellurene-based chemiresistive sensor devices and characterized them to address the morphology-dependent H_2_S gas detection properties of the resultant sensor devices.

## 2. Materials and Methods

### 2.1. Synthesis of Tellurene

We synthesized tellurene using a hydrothermal method as explained below. First, 4.4 mmol of PVP (Sigma Aldrich, St. Louis, MO, USA) was fully dissolved in 50 mL deionized water. PVP of different molecular weights (M.W. 58,000 or 360,000 g/mol) were incorporated, following the addition of 0.45 mmol of sodium tellurite (Na_2_TeO_3_, Sigma Aldrich, 99%) to the solution. As the components fully dissolved, the solution became transparent. Hydrazine (N_2_H_4_, Sigma Aldrich, 98%) and ammonium hydroxide solution (NH_4_OH, 28% NH_3_, Sigma Aldrich) were added as the reducing agent and pH adjustment, respectively. The dissolved solution was maintained for 10 min. The resulting solution was transferred to a Teflon-lined autoclave and heated at 180 °C for 30 h. After the reaction was completed, the Teflon-lined autoclave was left to cool down to room temperature. The obtained tellurene solution was washed four times with deionized water by centrifugation, and then the cleaned solution was dispersed in ethyl alcohol (Samchun Chemical Ltd., 99%, Seoul, South Korea).

### 2.2. Fabrication of Tellurene-Based Chemiresistive Gas Sensors

We fabricated the tellurene chemiresistive gas sensor devices using the drop-casting method. The as-synthesized tellurene solution was redispersed using a vortex mixer to ensure good dispersion. Then, 10 μL of the tellurene suspension was drop-casted onto a Si/SiO_2_ substrate with 200 nm thick-Au interdigitated electrodes made up a 50 μm spacing gap, and dried in an oven at room temperature for 30 min to fully remove the solvent.

### 2.3. Characterization

The morphologies of tellurene were investigated using optical microscopy (OM). The crystal structures and physical properties were observed through X-ray diffraction (XRD, D8 ADVANCE, Bruker, Billerica, MA, USA) with Cu Kα radiation and Raman spectroscopy (DXR2xi, Thermo Fisher Scientific Inc., Waltham, MA, USA) with a wavelength of 532 nm and an incident laser power of 6.1 mW. The transmission electron microscopy (TEM, Titan Themis Z, FEI, Hillsboro, USA) measurement was carried out to analysis the crystal structure and morphologies of the obtained samples. The electrical and gas-sensing properties were measured using a mini gas sensor chamber system (MPS-PT, Nextron, Daejeon, South Korea) connected to a source meter (2400, Keithley, Cleveland, OH, USA) to record the change in current or resistance in real-time. H_2_S and dry air were used as the target and standard base gases, respectively. Gas flow was modulated via mass flow controllers (TN-280SAV, Tylan Inc., San Diego, CA, USA) and 200 sccm of total flow rate was set for measurement. To attain stable gas-sensing characteristics, we evacuated all sensor devices in a vacuum chamber at room temperature for 30 min before performing the gas-sensing measurement.

## 3. Results and Discussion

**Figure 1:**
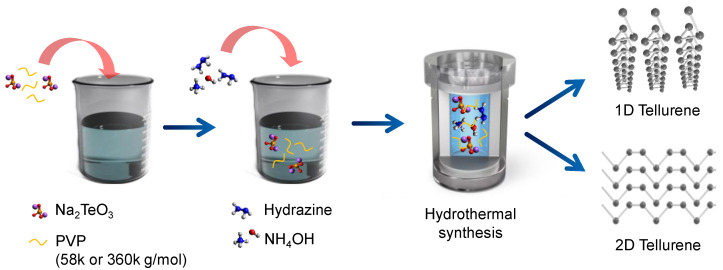
Schematic illustration of the synthesis procedure of tellurene via the hydrothermal route. PVP of different molecular weights was used to produce different morphologies of tellurene (1D or 2D).

Figure 1 illustrates the overall synthesis procedure of tellurene via the hydrothermal approach. Briefly, we first dissolved PVP in deionized water; PVP of different molecular weight (58 k or 360 k g/mol) was used to change the morphology of tellurene. Afterward, Na_2_TeO_3_ (Te precursor) was added to the solution, which was left to stand for 10 min. Hydrazine and NH_4_OH were then added to this solution, and the fully dissolved solution was transferred to a Teflon-lined autoclave. The autoclave was heated at 180 °C for 30 h, and the obtained products were washed with deionized water.

**Figure 2:**
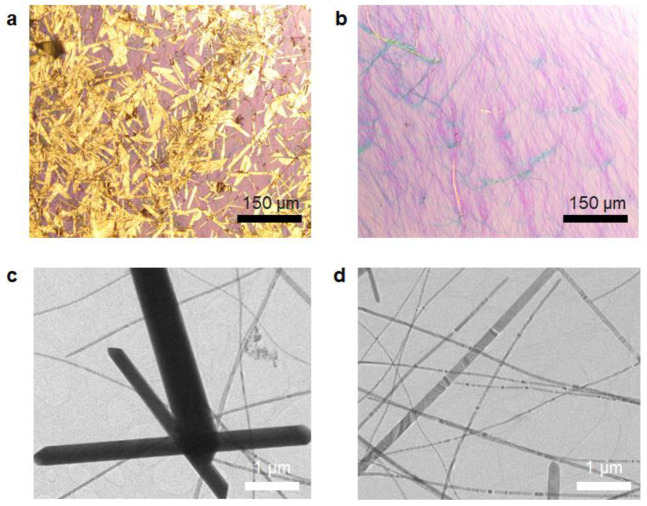
OM images of tellurene synthesized using PVP with molecular weights of (**a**) 58 k and (**b**) 360 k g/mol via the hydrothermal method. TEM images of tellurene synthesized using PVP with molecular weights of (**c**) 58 k and (**d**) 360 k g/mol.

In hydrothermal synthesis, the morphologies of the obtained samples can be affected by various process parameters, including the reaction time, temperature, and surfactant [19,20]. Among them, the molecular weight of the surfactant strongly induces changes in the morphologies of the samples because it strongly influences the inherent adsorption properties on the specific crystal facet [21]. From this point, we used PVP of both molecular weights (58 k and 360 k g/mol) to determine their effects on growth while applying the same synthesis protocol (Figure 1 and Figure 2). Two-dimensional flakes (average width: 6.49 µm and length: 41.8 µm) with a little presence of nanowires were observed when using PVP with a molecular weight of 58 k g/mol (Figure 2a). A 1D form with an average width of 53 nm and length of 56.7 µm were only obtained when using PVP with a molecular weight of 360 k g/mol (Figure 2b). This suggests that the molecular weight of PVP is strongly associated with the morphology of tellurene. TEM measurement was also performed to clearly observe the morphology of samples obtained using PVP with molecular weights of 58 k and 360 k g/mol (Figure 1c,d). The sample prepared with PVP of 58 k g/mol predominantly exhibited a thick and flake-like structure with a little presence of a wire-like one, whereas the sample obtained using PVP of 360 k g/mol predominantly showed nanobelts form, but not a nanowire-like one. In general, tellurene tends to grow anisotropically along the 1D direction [12], but PVPs are favorably adsorbed on the (100) facet [21], which contributes to vertical growth [12]. The preferential chemisorption on the Te (100) facet prevents the diffusion of Te atoms, leading to the 2D growth of tellurene rather than vertical growth. This favorable growth direction can be supported by the selected area diffraction pattern (SAED, Figure S1). We clearly observed the clear atomic arrangement, and thus, the perfect single crystal pattern appeared. In addition, the interplanar distance was estimated to be approximately 0.6 nm. It is indicative of the growth along the [001] direction.

The hydrophobic chains (alkyl group) of the PVP with the higher molecular weight (360 k g/mol) were longer than those of the PVP with the lower molecular weight (58 k g/mol); the lengths were estimated to be approximately 576 and 92 nm, respectively [21]. These longer hydrophobic chains introduced a strong repulsive force between the adjacent PVP molecules, which exhibited a greater 2D growth. Thanks to this unusual growth mechanism, 1D Te exhibited a 2D-like nanobelt one, and similar results were also reported by Wang et al. They also demonstrated that tellurene grew along the 1D direction when PVP with a higher molecular weight was used [21]. Hereafter, the samples synthesized using PVP with molecular weights of 58 k and 360 k g/mol are referred to as 2D Te and 1D Te, respectively.

**Figure 3:**
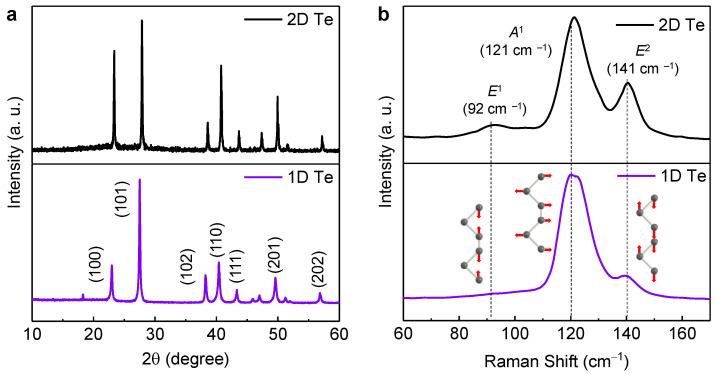
(**a**) XRD patterns and (**b**) Raman spectra of 1D and 2D tellurene synthesized using PVP with molecular weights of 58 k and 360 k g/mol, respectively.

Figure 3 displays the XRD patterns of the 1D and 2D Te. All samples presented identically prominent peaks located at 23°, 27.6°, 38.3°, 40.4°, 43.3°, 49.6°, 56.9°, and 63.8°, which correspond to the (100), (101), (102), (110), (111), (201), (202), and (210) planes in the hexagonal Te structure, respectively (JCPDS No. 36-1452). There were no distinct impurity peaks and full-width-half-maximums of the main peaks were narrow, indicating that a high-quality Te structure was attained. However, there was a slight difference in the relative intensities of the (100), (102), and (110) planes for 2D and 1D Te. The PVP-dependent specific crystal growth mechanism of tellurene remains unclear, but the stronger (100) peak in the 2D Te sample, synthesized using PVP with a molecular weight of 58 k g/mol, implies that 2D directional growth is associated with {10$\bar{1}$0} planes. This is in line with a previous report [12,21]. Thanks to a 2D direction growth behavior introduced by PVP, 1D Te seems like it has a nanobelt form in Figure 1, as mentioned above.

Raman spectroscopy measurements were also performed to further confirm how the inherent structural vibration modes change depending on the Te forms (Figure 3b). Two-dimensional Te showed three dominant peaks at 92, 121, and 141 cm^–1^, which correspond to the E_1_, A_1_, and E_2_ modes, respectively [22]. This implies that all vibration modes are consistent with those in the literature [12,21,23]. Interestingly, the E_1_ vibration mode disappeared in the 1D Te sample. The E_1_ mode is associated with the bond-bending vibration along the $\left[\bar{1}2\bar{1}0\right]$ direction, leading to lateral growth in Te [21]. This phenomenon further confirms that the change in the morphology of Te is well aligned with OM images (Figure 2).

**Figure 4:**
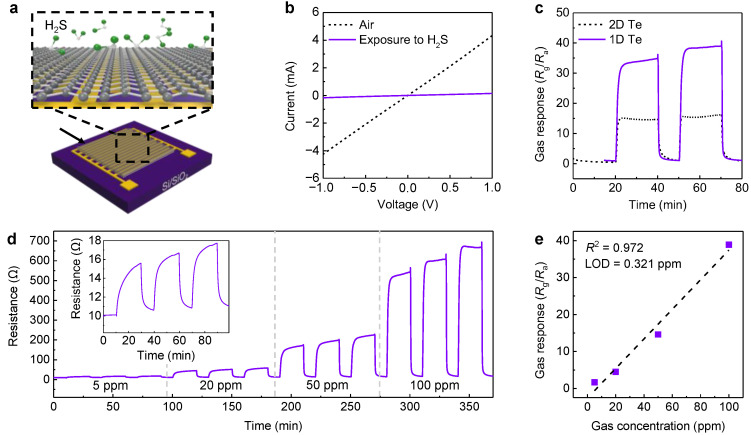
(**a**) Schematic illustration showing the Te-based chemiresistive gas sensor device. The solution containing Te was drop-casted onto interdigitated electrodes on a Si/SiO_2_ substrate. (**b**) I–V characteristics of tellurene chemiresistive sensors under air or exposure to 100 ppm H_2_S. (**c**) Two-step dynamic gas-sensing characterizations of tellurene sensors depending on the morphologies under exposure to 100 ppm H_2_S. (**d**) Dynamic gas-sensing characterization of the 1D Te sensor depending on the gas concentration (5–100 ppm). The inset graph indicates gas-sensing characterizations under consecutive exposure to 5 ppm H_2_S. (**e**) Gas response of the 1D Te-based sensor as a function of gas concentration.

We drop-casted the solution containing Te samples onto Si/SiO_2_ substrates with 200 nm-thick Au interdigitated electrodes to explore the morphology-dependent H_2_S response, as shown in Figure 4a. To prevent the side effect issue, all chemiresistive sensor devices were evacuated in a vacuum chamber for 30 min. Figure 4b presents the I–V characteristics of Te sensor devices before and after exposure to 100 ppm H_2_S. Under air, a clear linear relationship between the voltage and current was observed, implying that Ohmic contact was well formed between the channels and metal contacts. Au is a high-work function metal, and tellurene generally shows the *p*-type behavior [12]. In case of that, the Schottky barrier height is lower when using low-work function metals, including titanium or silver, due to the significant differences between their Fermi levels. If the low-work function metal is employed, the non-linear behavior (Schottky contacts) can be observed, which would affect the sensing behavior of the resulting devices. Interestingly, a drop in the current level occurred upon exposure to 100 ppm H_2_S, indicating that a charge transfer between H_2_S and the tellurene channels occurred. Tellurene typically shows the *p*-type behavior, and H_2_S is a reducing gas that serves as an electron donator, which allows for a decrease in the majority hole carriers in tellurene. This interaction results in a decrease in the current level [16]. Figure 4c shows the dynamic gas-sensing characteristics of tellurene sensors fabricated using the 1D and 2D Te samples. All samples exhibited a clear gas response and stable saturation behaviors accompanied with a good reversibility when exposed consecutively to 100 ppm H_2_S.

To quantitatively calculate the gas-sensing performance, we defined the gas response as follows [24,25]:

Gas response = R_g_/R_a_,

where R_g_ and R_a_ are the resistances under air (base gas) and the target gas (H_2_S), respectively. It is noteworthy that the 1D Te-based device showed a higher gas response (~38) than the 2D Te-based one (~15) at room temperature. This remarkable performance could be attributed to the larger specific surface area of the 1D Te device compared to that of the 2D Te device. To investigate effect on a high operating temperature, we conducted the dynamical gas sensing characterization at an elevated temperature of 100 °C (Figure S3). The achieved gas response to 100 ppm H_2_S at this higher temperature was approximately 4.5, which was lower rather what was obtained at room temperature. This suggests that H_2_S gas molecules may have a tendency to desorb easily from the surface of tellurene, in contrast to metal oxides that typically require temperatures exceeding 300 °C for effective operation [4,13]. This result indicates that the optimal sensing temperature is room temperature. Response time can be defined as the time it takes for the chemiresistive sensor devices to reach 90% of the total resistance change. Recovery time is also defined as the time required for the sensors to return to 10% of their initial resistance. All devices exhibited fast responses and recovery times, which were estimated to be approximately 110 and 60 s, respectively. In previous reports on the chemiresistive gas sensor, based on 2D black phosphorus, similar to tellurene, the response time and recovery time was significantly slower (a few minutes) than those of our tellurene devices. This outstanding performance was ascribed to a good stability and a high inherent conductivity due to the high crystallinity of the resultant tellurene.

We performed H_2_S gas-sensing characterizations of the 1D Te-based sensor depending on the H_2_S gas concentration (5–100 ppm) (Figure 4d). As the gas concentration increased, the baseline resistance was well maintained in a vicinity below 50 Ohm. Most chemiresistive gas sensor devices based on 2D materials have exhibited the unstable baseline resistance and high baseline resistances level (a few MOhm) due to high defect concentration. In contrast, our 1D Te showed the single crystal lattice structure (Figure S1) coupled with relatively higher crystallinity. This observation indicates a lower inherent defect concentration, compared to that of conventional TMD materials. In addition, the gas response gradually increased, and a linear relationship was observed while preserving a clear gas response in the whole range. Over the whole concentration range, the correlation coefficient (R^2^) was estimated to be approximately 0.972, which indicates the significant linearity in the vicinity of ppm-level H_2_S concentrations. Such high linearity results in an outstanding limit of detection (LOD; 0.3 ppm) [26], revealing that our 1D Te-based sensor would be also capable of sub-ppm H_2_S detection. 

**Figure 5:**
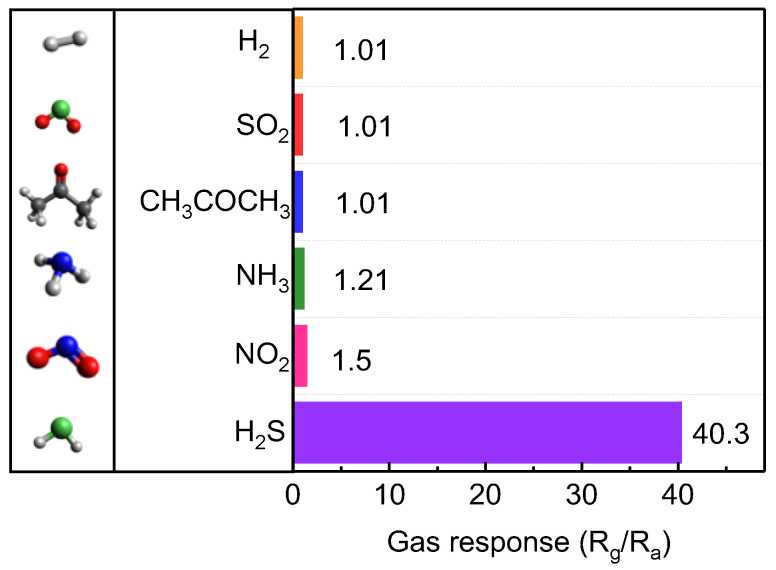
Gas selectivity of the 1D Te sensor under exposure to 100 ppm H_2_S, NO_2_, NH_3_, CH_3_COCH_3_, SO_2_, and H_2_.

We also characterized the gas selectivity of the optimal 1D Te-based sensor by comparing the responses to various gas molecules, including NO_2_, CH_3_COCH_3_, SO_2_, NH_3_, and H_2_ (Figure 5 and Figure S2). Despite exposure to an identical gas concentration (100 ppm), our sensor exhibited the highest gas response to H_2_S (40 times higher than that to other gas molecules) (Figure 5). This superior gas selectivity originated from the high crystallinity of the obtained Te and the strong adsorption energy between the Te atoms and H_2_S gas molecules [17].

Table 1 summarizes the comparison of characterizations and performances of our 1D Te samples and Te-based gas sensors reported previously. Most tellurium-based gas sensors exhibited higher NO_2_ gas responses, while our tellurene gas sensors achieved a lower NO_2_ gas response. This noticeable distinction would be presumably due to the predominant facets that are grown. Yuan et al. demonstrated an Au-loaded tellurium gas sensor for NO_2_ detection [27]. They conducted density functional of theory (DFT) calculation, revealing that the preferential adsorption site for NO_2_ molecules is (100) facet. This suggests that a higher intensity of (100) peak is associated with a higher NO_2_ gas response. In contrast, our 1D Te samples exhibited relatively lower (100) peak intensities. This indicates the potential for distinct gas interaction behavior. This finding can be also supported by Wang et al. [16], where they also conducted tellurene-based gas sensing characterizations. In their samples, (100) intensity was also lower than (101) intensity than that of (101), leading to a higher H_2_S gas response, similar to what we observed in our results. For the 2D Te sample, (100) peak intensity was comparatively higher than that of 1D Te because of 2D directional growth, yet it still remained lower than that of the (101) plane. This trend in relative peak intensities contributed to similar selectivity’s observed the 2D Te sample (Figure S4).

**Figure 6:**
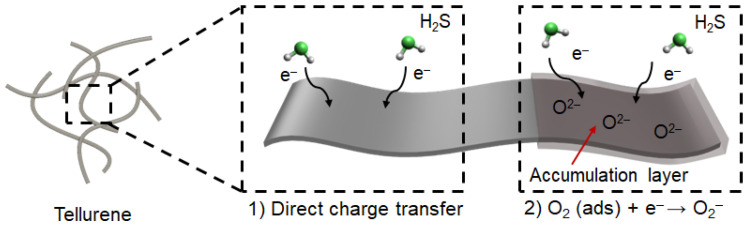
Schematic images of the 1D Te structure and H_2_S gas-sensing mechanism of tellurene at room temperature.

As mentioned above, tellurene is a van der Waals material, and its gas-sensing mechanism mostly complies with the direct charge transfer between atoms and gas molecules (Figure 6). This mechanism relies on the specific surface area of the obtained sensing materials [19], which is associated with the number of active sites, compared to that which works by depletion layer. Moreover, oxygen molecules in the air are physically adsorbed on the surface of Te at room temperature, resulting in a thin accumulation layer that further improves the H_2_S gas response (Figure 6) [16].

O_2_ (ads) + e^−^ ↔ O_2_^−^ (30 °C, physisorption)

The formed oxygen ions on the surface of tellurene additionally interacts with H_2_S gas molecules, leading to further enhancement in H_2_S gas response (Figure 6). Thanks to these working principles, the 1D Te-based chemiresistive gas sensor device showed the higher gas response, compared to that of the 2D Te-based one.

## 4. Conclusions

We tailored the morphologies of tellurene using PVP with different molecular weights via the hydrothermal method. Depending on the molecular weight of PVP, 1D or 2D Te was formed while preserving high crystallinity. This distinct control depends on the favorable adsorption properties of PVP on the specific crystal plane of Te. We fabricated tellurene-based chemiresistive sensor devices. One-dimensional Te chemiresistive sensor devices presented superior H_2_S gas detection properties compared to the 2D Te one, even achieving a gas response to H_2_S of ~38. This value was nearly 40 times higher than that to other gas molecules. Moreover, relatively faster response time and recovery time of 110 s and 60 s, respectively, were achieved compared to those of TMD-based chemiresistive gas sensor devices. These improvements can be attributed to the high H_2_S gas adsorption energy and the high inherent carrier mobility coming from the single crystal of tellurene. Our findings demonstrate how to fabricate effective chalcogen-based gas sensors and enhance their gas detection properties at room temperature.

## Figures and Tables

**Table 1:** Comparison of gas sensing performances and main diffraction peaks of our 1D Te sensors and Te-based gas sensors reported previously.

Structure	Synthesis Method	H_2_S Response (R_g_/R_a_)	NO_2_ Response (R_g_/R_a_)	Main Diffraction Peak in XRD	Ref.
2D Te	Hydrothermal	-	2.22 (1 ppm)	(100)	[13]
1D Te	Chemical vapor deposition	-	1.30 (0.2 ppm)	(100)	[14]
2D Te	Hydrothermal	5.6 (10 ppm)	-	(101)	[16]
Au- 2D Te	Hydrothermal	1.13 (1 ppm)	3.18 (0.1 ppm)	(100)	[27]
1D Te	Liquid exfoliation	-	3.4 (0.1 ppm)	(100)	[28]
1D Te	Hydrothermal	40.3 (100 ppm)	1.5 (100 ppm)	(101)	This work

## Supplementary Materials

The following supporting information can be downloaded at: https://www.mdpi.com/article/10.3390/nano13192707/s1, Figure S1: High-resolution TEM image of the obtained tellurene using PVP with a molecular weight of 360k g/mol. Inset image indicates SAED pattern of tellurene; Figure S2: (a) Gas-sensing characterizations of the 1D Te sensor under exposure to H_2_S, NO_2_, NH_3_, CH_3_COCH_3_, SO_2_, and H_2_. (b) Enlarged plot of gas sensing characterization of the 1D Te sensor under exposure to to 100 ppm of H_2_S, NO_2_, NH_3_, CH_3_COCH_3_, SO_2_, and H_2_; Figure S3: Dynamic gas sensing characterizations of 1D Te-based sensors under exposure to 100 ppm H_2_S. Operating temperature is 100 °C; Figure S4: Dynamic gas sensing characterizations of 2D Te-based sensor under exposure to 100 ppm NO_2_ and NH_3_.
