# Supplementary material for: Controlling the Morphology of Tellurene for a High-Performance H2S Chemiresistive Room-Temperature Gas Sensor
Source: Nanomaterials (Basel). 2023 Oct 5;13(19):2707. doi: 10.3390/nano13192707 (PMC10574203; doi:10.3390/nano13192707)
Supplement: Supplementary file 1 [file nanomaterials-13-02707-s001.zip › nanomaterials-2608401-supplementary.pdf]

Supplementary Materials

Controlling Morphology of Tellurene for a High-Performance H_2S Chemiresistive Room-Temperature Gas

Yeonjin Je[1,2] and Sang-Soo Chee[1,*]

[1]Nano Convergence Materials Center, Korea Institute of Ceramic Engineering and Technology (KICET), Jinju, 52851, Republic of Korea

[2]Department of Materials Science and Engineering, Korea University, Seoul, 02841, Republic of Korea

*Correspondence: sschee@kicet.re.kr (S.S.C.)

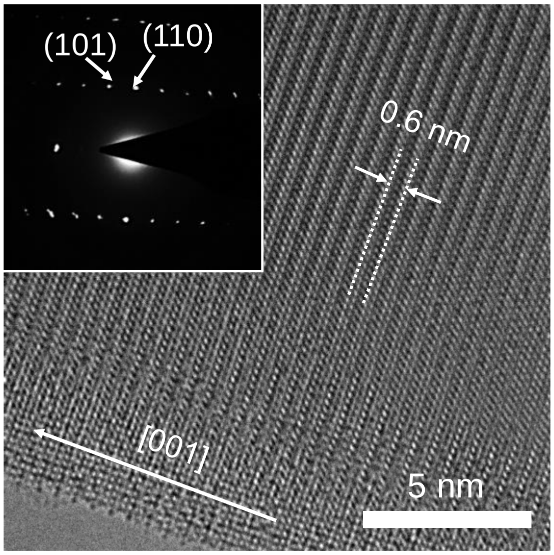

Figure S1. High-resolution TEM image of the obtained tellurene using PVP with a molecular weight of 360k g/mol. Inset image indicates SAED pattern of tellurene.

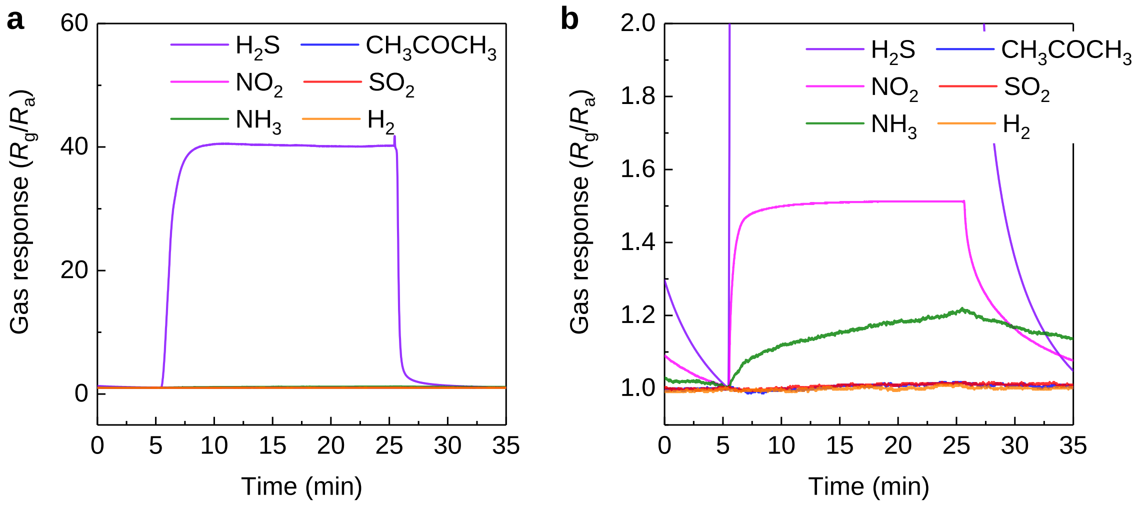

Figure S2. (a) Gas-sensing characterizations of the 1D Te sensor under exposure to H_2S, NO_2, NH_3, CH_3COCH_3, SO_2, and H_2. (b) Enlarged plot of gas sensing characterization of the 1D Te sensor under exposure to to 100 ppm of H_2S, NO_2, NH_3, CH_3COCH_3, SO_2, and H_2.

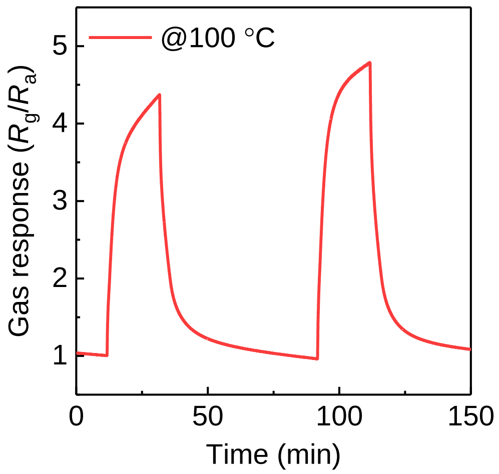

Figure S3. Dynamic gas sensing characterizations of 1D Te-based sensors under exposure to 100 ppm H_2S. Operating temperature is 100 °C.

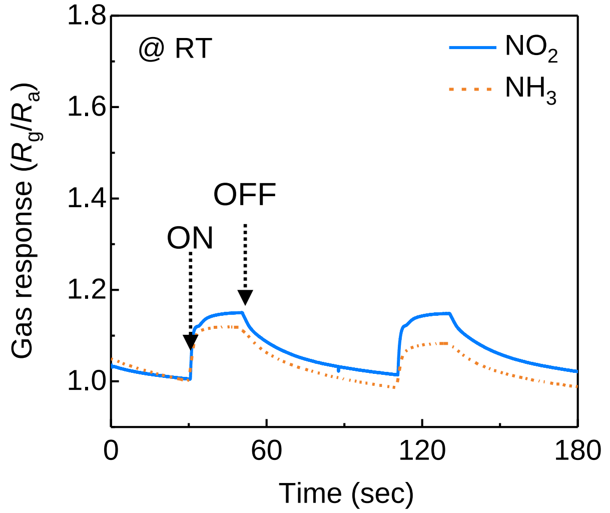

Figure S4. Dynamic gas sensing characterizations of 2D Te-based sensor under exposure to 100 ppm NO_2 and NH_3.
